# Quantifying vitamin D intake among Aboriginal and Torres Strait Islander peoples in Australia

**DOI:** 10.1038/s41430-025-01580-7

**Published:** 2025-02-19

**Authors:** Belinda Neo, Dale Tilbrook, Noel Nannup, Alison Daly, Eleanor Dunlop, John Jacky, Carol Michie, Cindy Prior, Brad Farrant, Carrington C. J. Shepherd, Anita S. Lawrence, Edoardo Tescari, Lucinda J. Black

**Affiliations:** 1 Curtin Medical School, Curtin University, Bentley, Western Australia Australia; 2 Maalinup Aboriginal Gallery, Caversham, Western Australia Australia; 3 The Kids Institute, The University of Western Australia, Nedlands, Western Australia Australia; 4 Curtin School of Population Health, Curtin University, Bentley, Western Australia Australia; 5 Institute for Physical Activity and Nutrition (IPAN), School of Exercise and Nutrition Sciences, Deakin University, Geelong, Victoria Australia; 6 Ngangk Yira Institute, Murdoch University, Murdoch, Western Australia Australia; 7 School of Agriculture and Food, and Ecosystem Sciences, The University of Melbourne, Parkville, Victoria Australia; 8 Melbourne Data Analytics Platform, The University of Melbourne, Parkville, Victoria Australia

**Keywords:** Nutrition, Public health

## Abstract

**Background/Objective:**

Vitamin D deficiency (serum 25-hydroxyvitamin D [25(OH)D] concentration < 50 nmol/L) is prevalent among Aboriginal and Torres Strait Islander peoples in Australia. Alternative to sun exposure (the primary source of vitamin D), vitamin D can also be obtained from food (e.g. fish, eggs, and meat) and supplements. However, the vitamin D intake of this population group is unknown. We aimed to provide the first quantification of vitamin D intake using nationally representative data from Aboriginal and Torres Strait Islander peoples.

**Methods:**

We used food consumption data collected in the 2012–2013 National Aboriginal and Torres Strait Islander Nutrition and Physical Activity Survey (*n* = 4109) and vitamin D food composition data to quantify vitamin D intake by sex, age group, and remoteness of location. Wilcoxon rank-sum test was used to assess the difference in vitamin D intake between sexes and remoteness of location.

**Results:**

The median (25th, 75th percentile) vitamin D intake among Aboriginal and Torres Strait Islander peoples aged ≥ 2 years was 80 (45, 145) IU/day. Vitamin D intake was statistically significantly different between males and females (*p* = < 0.001). There was no statistically significant difference between vitamin D intake in non-remote and remote areas (*p* = 0.292).

**Conclusions:**

Vitamin D intake among Aboriginal and Torres Strait Islander peoples is low. Food-based public health strategies guided by Aboriginal and Torres Strait Islander Elders and communities could be developed to promote higher vitamin D intake among this population.

## Introduction

The primary source of vitamin D is ultraviolet-B radiation from sun exposure. Vitamin D obtained from sun exposure has not been measured among Aboriginal and Torres Strait Islander peoples [1]. However, the high prevalence of vitamin D deficiency (serum 25‐hydroxyvitamin D [25(OH)D] concentration <50 nmol/L) among Aboriginal and Torres Strait Islander peoples (27% of adults aged ≥ 18 years, 39% in those living remotely in Australia) [1] suggests that sun exposure is inadequate to ensure sufficient vitamin D status at the population level. Vitamin D deficiency may result in adverse health outcomes in musculoskeletal health [2] and non-communicable diseases (e.g. type 2 diabetes and cardiovascular diseases) which are highly prevalent among Aboriginal and Torres Strait Islander peoples [3, 4].

When sun exposure is minimal, dietary vitamin D intake is crucial to prevent vitamin D deficiency. The estimated average requirement for vitamin D intake recommended by the United States Institute of Medicine (assuming minimal sunlight exposure) is 400 IU/day for people aged ≥ 1 years [5]. Recently, four D vitamers were measured in retail food products and game products to develop Australia’s first comprehensive national vitamin D food composition database [6, 7]. Using those composition data, we previously quantified vitamin D intake in the general Australian population which included Aboriginal and Torres Strait Islander peoples except for those living in very remote areas and discrete communities [8]. Due to the small percentage of Aboriginal and Torres Strait Islander peoples included in that survey, sub-group analyses of that population group could not be conducted.

The 2012–2013 National Aboriginal and Torres Strait Islander Nutrition and Physical Activity Survey (NATSINPAS) was the first national survey to collect detailed national benchmark nutrition information among Aboriginal and Torres Strait Islander peoples. The NATSINPAS, in combination with our existing comprehensive vitamin D composition data, enabled us to quantify vitamin D intake using nationally representative data from Aboriginal and Torres Strait Islander peoples for the first time.

## Methods

### **Study population**

The NATSINPAS was conducted between August 2012 and July 2013 and provided the most recent food consumption data among Aboriginal and Torres Strait Islander peoples aged ≥ 2 years in Australia. Detailed methods can be found elsewhere [9]. Briefly, the survey was conducted across all states and territories in Australia. The remoteness of the location of residence was divided into two categories, remote and non-remote, based on the Australian Statistical Geography Standard classification [9]. From 3661 households that were approached, 2900 (79%) provided adequate responses. The total number of participants was 4109, with 1792 living in non-remote areas and 2317 living in remote areas.

The first survey interview was conducted face-to-face by a trained interviewer. An adult household member (aged ≥ 18 years) responded on behalf of those aged < 15 years, and on behalf of those aged 15–17 years who did not have parental consent to respond independently. Participants living in non-remote areas were invited to complete a second interview via telephone 8 days after the first interview.

The response rate for the first interview was 99.5%, and missing data for the remaining 0.5% were imputed by the Australian Bureau of Statistics [10]. The second interview was only conducted among participants living in non-remote areas (*n* = 771). Hence, we were unable to estimate usual vitamin D intake in remote areas.

### Food consumption data

A 24-h dietary food recall was conducted in the 2012–2013 NATSINPAS to collect detailed food consumption data from Aboriginal and Torres Strait Islander peoples aged ≥ 2 years. Detailed methods are reported elsewhere [9] and briefly summarised here. For the 24-h food recall interview segment, children aged 6–14 years were encouraged to participate with an adult household member present, rather than engaging an interview proxy [10]. For those aged 15–17 years, 61% were interviewed individually, and 39% were interviewed with an adult household member.

The Automated Multiple-Pass Method was developed by the United States Department of Agriculture [11]. It was adapted for use in Australia by Food Standards Australia New Zealand and integrated into the Computer-Assisted Personal Interview instrument [9]. Participants were asked systematic questions based on the Automated Multiple-Pass Method prompts, designed to accurately capture detailed information about the food, cooking methods, and portion sizes they consumed from midnight to midnight on the day before the interview. The Australian Health Survey (AHS) food model booklet and bush tucker prompt cards were used to help participants estimate the amount of food and beverages consumed [9]. Food consumption data were subsequently coded using the AHS classification system, an eight-digit numeric food identification code [12].

### Vitamin D composition data

We published vitamin D composition data for four D vitamers (vitamin D_3_, 25(OH)D_3_, vitamin D_2_, and 25(OH)D_2_) in retail food products and game products with detailed methods [6, 7]. Game products form part of the traditional diets of Aboriginal and Torres Strait Islander peoples [13, 14]. Briefly, to test for the presence of D vitamers, food products with potential to contain vitamin D were sampled across three Australian cities (Sydney, Melbourne, and Perth) [7]. We sampled 98 retail food products from 896 primary samples, which were analysed as 149 composite samples. We sampled 36 game meats including camel, crocodile, emu, and kangaroo from Victoria, and 8 emu eggs and 400 ml emu oil from emu farms in Western Australia, Victoria, and New South Wales [6]. The D vitamers in these foods were measured using liquid chromatography with triple quadrupole mass spectrometry at the National Measurement Institute of Australia, a laboratory accredited by the National Association of Testing Authorities to measure vitamin D in food (ISO17025:2017).

The Australian Food and Nutrient (AUSTNUT) Database 2011–2013 is a food nutrient database used to estimate nutrient intake from the AHS [12]. We measured vitamin D in 118 foods and obtained label data for 21 foods, and mapped those vitamin D concentrations to the AUSNUT database, following the method used for the Australian Total Diet Studies [15, 16]. We were able to map foods with detectable concentrations of vitamin D to 3680 of the 5740 foods in the AUSNUT database [6, 7, 12].

### Bioactivity of 25(OH)D

Biological activity refers to the bioaccessibility (the amount of nutrient potentially available for absorption) and bioavailability (the amount of nutrient absorbed that is available to be used and stored) of the nutrient [17]. A bioactivity factor of 1 assumes equal biological activity of vitamers. Among the four D vitamers, the hydroxylated D vitamers (25(OH)D_2_ and 25(OH)D_3_) may have higher biological activity compared to vitamin D_2_ and vitamin D_3_ [18, 19]. Although more recent evidence indicate that 25(OH)D is 2.5 times more bioactive compared to vitamin D [20], earlier evidence suggested that it was up to five times more bioactive. As the Australian Food Composition Database uses a bioactivity factor of 5 based on that earlier evidence, we quantified vitamin D intake using bioactivity factors 1 and 5 for 25(OH)D.

### Quantifying absolute vitamin D intake

We quantified the percentiles (5th, 25th, 50th, 75th, and 95th) for absolute vitamin D intake using day 1 of 24-h food recall data from NATSINPAS. The weight of each food consumed by each respondent per day (g) was multiplied by the vitamin D content of the food (μg/g). We presented the vitamin D intake data in IU using the following conversion, 1 μg = 40 IU. As remote areas were oversampled for this survey, the results were weighted to the benchmark of Aboriginal and Torres Strait Islander estimated resident population living in private dwellings of Australia on 30 June 2011, based on the 2011 Census of Population and Housing [9], with survey weight provided by the Australian Bureau of Statistics. Statistical analysis was performed using Stata version 17 (StataCorp, College Station, Texas, USA). The results were presented by sex, age group, and remoteness of location.

### Modelling usual vitamin D intakes in non-remote areas

The National Cancer Institute method requires at least 2 days of 24-h food recall data to address the within-subject variation of dietary intake [21]. The 24-h food recall was collected for 2 days for those living in non-remote areas, and one day for those living in remote areas. For participants living in non-remote areas, we calculated usual vitamin D intake using the National Cancer Institute method [21, 22]. The results are presented by sex and age group.

### Percentage of food group contribution to vitamin D intake

The percentage contribution of food groups according to the AHS classification system [12] to vitamin D intake was calculated as (total vitamin D intake from food group divided by total vitamin D intake from all foods) x 100 [23]. The top 10 food group contributors to total vitamin D intake were reported.

### Statistical analysis

The normality of absolute vitamin D intake data was visually examined by plotting a frequency distribution graph. As the data were non-parametric, we used an unweighted Wilcoxon rank-sum test to determine between-group differences in vitamin D intake between sexes and remoteness of location; *p* value of <0.05 indicated statistical significance.

## Results

### Bioactivity factor 1

Across all ages for both sexes, the median (25th, 75th percentile) vitamin D intake was 80 (45, 145) IU/day **(**Table 1**)**. Older adults aged ≥ 71 years had the lowest median vitamin D intake, and children aged 9–13 years had the highest median vitamin D intake. The vitamin D intake in males was statistically significantly different compared to females (*p* = <0.001). Older female adults aged ≥ 71 years had the lowest median vitamin D intake at 41 IU/day, and male children aged 9–13 years and adults 31–50 years had the highest median (25th, 75th percentile) vitamin D intake at 101 (67, 169) and 101 (60, 178) IU/day, respectively.

**Table 1:** Vitamin D intake of Aboriginal and Torres Strait Islander peoples, stratified by sex and age group.

Age group			Bioactivity factor 1					Bioactivity factor 5^b^				
			Percentile (IU/day)					Percentile (IU/day)				
(years)	*n*	*n*^a^	5th	25th	50th	75th	95th	5th	25th	50th	75th	95th
Total												
All ages	4109	606915	14	45	80	145	322	36	98	167	271	521
2–3	240	31011	14	31	49	83	222	43	81	122	171	378
4–8	515	82691	15	43	73	120	292	36	89	142	219	487
9–13	409	70929	16	53	93	161	356	43	116	182	264	472
14–18	332	68823	10	37	76	153	331	26	86	164	285	568
19–30	722	130568	17	50	89	160	353	44	110	183	321	553
31–50	1098	146120	15	51	89	152	327	35	108	176	294	525
51–70	722	70659	13	45	78	135	338	34	90	165	270	521
≥71	71	6114	13	33	41	97	275	37	85	108	172	364
Male												
All ages	1814	301992	16	52	89	163	348	40	114	183	304	551
2–3	125	16947	16	40	52	74	222	53	88	122	171	379
4–8	259	40953	12	46	72	142	289	32	91	139	242	487
9–13	207	37866	14	67	101	169	361	43	128	195	287	472
14–18	164	34093	12	47	94	159	328	35	109	201	321	594
19–30	299	66534	22	56	98	191	372	56	132	216	363	627
31–50	427	68967	19	60	101	178	348	49	143	198	327	622
51–70	308	34470	14	46	75	139	281	38	108	179	271	516
≥ 71	25	2162	25	36	64	162	496	43	90	122	227	619
Female												
All ages	2295	304923	12	39	74	126	292	31	87	147	245	492
2–3	115	14065	13	26	44	95	280	42	72	109	163	340
4–8	256	41737	17	41	73	114	292	39	85	145	212	491
9–13	202	33064	19	49	88	138	233	48	100	148	239	488
14–18	168	34730	8	27	57	114	356	21	67	116	256	557
19–30	423	64034	12	42	79	136	311	26	89	161	266	511
31–50	671	77153	11	40	78	132	271	28	90	157	255	477
51–70	414	36189	13	43	80	135	352	34	86	153	270	630
≥71	46	3951	10	33	41	92	141	35	81	96	132	239

^a^Weighted to the benchmark of Aboriginal and Torres Strait Islander estimated resident population living in private dwellings of Australia at 30 June 2011, based on the 2011 Census of Population and Housing, with survey weight provided by the Australian Bureau of Statistics.

^b^A bioactivity factor of 5 was used as 25‐hydroxyvitamin D may be up to five times more bioactive than vitamin D.

There was no statistically significant difference between vitamin D intake in non-remote and remote areas (*p* = 0.292) **(**Table 2**)**. Vitamin D intake for non-remote and remote areas by sex and age group are reported in Supplementary Tables 1 and 2, respectively.

**Table 2:** Vitamin D intake of Aboriginal and Torres Strait Islander peoples, stratified by the remoteness of location and age group.

Age group			Bioactivity factor 1					Bioactivity factor 5^b^				
			Percentile (IU/day)					Percentile (IU/day)				
(years)	*n*	*n*^a^	5th	25th	50th	75th	95th	5th	25th	50th	75th	95th
Non-remote												
All ages	1792	477901	15	45	80	146	318	38	98	167	268	518
2–3	102	24762	16	35	53	83	222	45	88	122	171	379
4–8	214	67058	15	46	74	122	292	39	89	142	219	491
9–13	176	56286	15	52	95	166	360	43	117	180	261	472
14–18	153	55473	11	36	76	153	328	23	82	169	284	568
19–30	332	102660	18	51	85	160	343	46	104	179	312	539
31–50	482	112355	15	52	91	150	292	35	108	178	294	513
51–70	300	54231	14	46	78	139	338	37	90	165	270	539
≥71	33	5076	22	33	41	97	275	43	86	105	167	343
Remote												
All ages	2317	129014	10	42	80	140	337	31	97	167	286	574
2–3	138	6249	7	22	41	73	159	26	62	103	145	265
4–8	301	15633	12	36	64	104	259	30	82	141	204	386
9–13	233	14644	17	54	89	125	267	54	109	197	266	456
14–18	179	13350	7	46	82	144	381	26	101	163	285	636
19–30	390	27909	11	49	102	167	385	34	116	202	335	641
31–50	616	33765	13	46	81	154	342	39	103	173	300	709
51–70	422	16427	9	42	78	121	299	26	94	162	278	488
≥ 71	38	1037	10	24	49	96	202	35	68	157	188	364

^a^Weighted to the benchmark of Aboriginal and Torres Strait Islander estimated resident population living in private dwellings of Australia at 30 June 2011, based on the 2011 Census of Population and Housing, with survey weight provided by the Australian Bureau of Statistics.

^b^A bioactivity factor of 5 was used as 25‐hydroxyvitamin D may be up to five times more bioactive than vitamin D.

Across the age groups and sexes, ‘Meat, poultry, game products and dishes’ had the highest contribution to total vitamin D intake, followed by ‘Fats and oils,’ and ‘Egg products and dishes’ (Table 3). Vitamin D intake from game meats was 5196 IU/day, which contributed 1.1% to total vitamin D intake. The top 10 food group contributors to total vitamin D intake for non-remote and remote areas are found in Supplementary Tables 3 and 4, respectively.

**Table 3:** Top 10 food group contributors to vitamin D intake among Aboriginal and Torres Strait Islander peoples.^a^.

Bioactivity factor 1	IU	%	Bioactivity factor 5^b^	IU	%
Meat, poultry, game products and dishes	97550	21.0	Meat, poultry, game products and dishes	281903	32.7
Fats and oils^c^	90156	19.4	Egg products and dishes	159923	18.6
Egg products and dishes	66245	14.2	Fats and oils^3^	93551	10.9
Fish and seafood products and dishes	65545	14.1	Biscuits, pastries, cakes, burgers, and pasta	85346	9.9
Biscuits, pastries, cakes, burgers, and pasta	47574	10.2	Milk products (e.g. milk, yoghurt, cheese, ice cream)	80838	9.4
Non-alcoholic beverages	32292	6.9	Fish and seafood products and dishes	68716	8.0
Milk products (e.g. milk, yoghurt, cheese, ice cream)	24065	5.2	Non-alcoholic beverages	35036	4.1
Confectionery and cereal/nut/fruit/seed bars	11863	2.5	Breakfast cereals and bread	13851	1.6
Breakfast cereals and bread	10436	2.2	Confectionery and cereal/nut/fruit/seed bars	12147	1.4
Vegetable products and dishes	4485	1.0	Vegetable products and dishes	7186	0.8

^a^Calculated as (total vitamin D intake from food group/total vitamin D intake from all foods) x 100.

^b^A bioactivity factor of 5 was used as 25‐hydroxyvitamin D may be up to five times more bioactive than vitamin D.

^c^Fats and oils food group includes margarine, table spreads, and butter.

### Bioactivity factor 5

When a bioactivity factor of 5 was applied, the median (25th, 75th percentile) vitamin D intake was 167 (98, 271) IU/day, and the highest median vitamin D intake was observed in adults aged 19–30 years at 183 IU/day **(**Table 1**)**.

Similar to bioactivity factor 1, older adults aged ≥ 71 years had the lowest median vitamin D intake. Vitamin D intake between males and females was statistically significantly different (*p* = <0.001). There was also no statistically significant difference in vitamin D intake between non-remote and remote areas (*p* = 0.415). The top three food group contributors to total vitamin D intake were ‘Meat, poultry, game products and dishes,’ ‘Egg products and dishes,’ and ‘Fats and oils’ (Table 3). Vitamin D intake from game meats was 10639 IU/day, which contributed 1.2% to total vitamin D intake.

### Usual vitamin D intakes for non-remote areas

Usual mean vitamin D intakes ranged between 70–159 IU/day for those living in non-remote areas **(**Supplementary Table 5**)**. Across all age and sex groups, usual vitamin D intake percentiles (5th, 25th, and 50th) were higher than absolute vitamin D intakes. When equal bioactivity was assumed, <1% of Aboriginal and Torres Strait Islander peoples living non-remotely had adequate vitamin D intakes when compared to the estimated average requirement of 400 IU/day recommended by the United States Institute of Medicine [5]. None had intakes that exceeded the Australian upper level of intake of 3200 IU/day [24], or the United States Institute of Medicine tolerable upper intake level of 2500 to 4000 IU/day [5].

## Discussion

Similar to the general Australian population [8], vitamin D intakes were low among Aboriginal and Torres Strait Islander peoples of all age groups and sexes for both bioactivity factors 1 and 5 for 25(OH)D. Our findings indicate that males had a higher vitamin D intake compared to females, similar to our previous analysis in the general Australian population [8]. The higher vitamin D intake among males may be due to the higher overall food intake compared to females [10]. Given the high prevalence of vitamin D deficiency [1] and low vitamin D intakes among Aboriginal and Torres Strait Islander peoples, evidence-based public health strategies are needed to promote vitamin D sufficiency.

Assuming equal bioactivity among D vitamers, ‘Meat, poultry, game products, and dishes’ had the greatest contribution to vitamin D intake among Aboriginal and Torres Strait Islander peoples, indicating that those foods are widely consumed in this population. Comparatively, ‘Fish and seafood products and dishes’ contributed the greatest to vitamin D intake in the general Australian population [8]. Given that edible oil spreads (e.g. margarine) are the only foods mandated for vitamin D fortification in Australia [25], it was not surprising that ‘Fats and oils’ were one of the top three food group contributors to vitamin D intake for both bioactivity factors 1 and 5 for 25(OH)D. Foods such as beef, chicken, and eggs have a higher concentration of 25(OH)D compared to other foods (e.g. margarine, fin fish with <5% fat, and seafood products) [7]. When a bioactivity of 5 for 25(OH)D was applied, ‘Meat, poultry, game products and dishes’ was the greatest contributor to vitamin D intake for Aboriginal and Torres Strait Islander peoples and the general Australian population [8].

Vitamin D intake almost doubled across all sex and age groups when we applied a bioactivity factor of 5. A bioactivity factor of 5 is used in some international food composition databases (e.g. United Kingdom and India) [26, 27], while others do not include 25(OH)D (e.g. United States and Canada), and equal bioactivity is assumed across all D vitamers [28, 29]. We used bioactivity factors of both 1 and 5 for 25(OH)D, which allows for the comparison of vitamin D intake with other countries. However, as there is no current international consensus on the bioactivity factor of 25(OH)D, it has been suggested that equal bioactivity should be assumed until more definitive results are available [17].

The data on vitamin D intake among Indigenous peoples worldwide are notably scarce, which might be attributable to a lack of nationally representative food consumption data for those population groups [30, 31]. Notable exceptions include subsets of populations such as the (i) Dené/Métis communities in Canada (mean vitamin D intake at 186 IU/day for those aged 7–85 years) [32]; (ii) lactating Inuit women in the Inuvialuit Settlement Region, Nunavut, and Nunatsiavut communities in Canada (mean (SD) vitamin D intake at 192 (208) IU/day for those aged > 18 years) [33]; (iii) Māori people in New Zealand (median (interquartile range) vitamin D intake at 128 (156) IU/day for those aged 80–90 years) [34]; and (iv) Sami people in northern Norway (median vitamin D intake at 444 IU/day for males and 376 IU/day for females aged 40–69 years) [35]. In line with our findings, vitamin D intake among the Indigenous peoples was predominantly low. Vitamin D intake quantified in these studies, however, may be underestimated as not all D vitamers were accounted for in the food composition data, especially 25(OH)D, which may have greater biological activity than vitamin D.

Low vitamin D intake among Indigenous peoples [32–35] could be due to the dietary transition of food preferences, where consumption of game products has declined [36]. Traditional foods are considered valuable sources of micronutrients such as vitamin D [30, 37]. However, colonisation, modernisation, and food insecurity have altered the food availability and choices of Indigenous people worldwide [30, 38, 39]. A similar transition has been seen in the diet of Aboriginal and Torres Strait Islander peoples since the colonisation of Australia began about 250 years ago and ultimately resulted in many Aboriginal and Torres Strait Islander peoples being forcibly relocated and denied access to traditional foods and land [38]. Traditional foods were replaced by food rations imposed through government policy, which were energy-dense, and high in sugar, sodium, and fat [38]. Hence, strategies to promote the cultivation and consumption of nutrient-rich traditional foods could be developed through co-designed research led by Elders and Aboriginal and Torres Strait Islander peoples, drawing upon their traditional knowledge of growing and preparing traditional foods. Elders are highly respected, influential individuals in their community with a wealth of traditional knowledge and experience in traditional foods [40]. Promoting traditional ways of eating may improve vitamin D intake and status among Aboriginal and Torres Strait Islander peoples.

Given the prevalence of vitamin D deficiency in Australia, which affects 27% of Aboriginal and Torres Strait Islander peoples aged ≥ 18 years [1] and 20% of general Australians aged ≥ 25 years [41], there is a need to explore population-based dietary approaches such as supplementation and food fortification to promote vitamin D sufficiency. For example, countries such as Canada and Denmark have recommended vitamin D supplementation for specific at-risk population groups (e.g. older adults) [42, 43]. However, potential limitations for implementing supplementation as a population-wide public health strategy include adherence and potential vitamin D toxicity [44]. There is currently no public health recommendation for vitamin D supplementation in Australia.

Alternatively, some countries have used food fortification of commonly consumed foods (e.g. milk, bread, and breakfast cereals) as a public health strategy to increase vitamin D intake [45–47]. For example, after vitamin D food fortification was implemented in Finland, mean vitamin D intake increased from 200 to 680 IU/day for males and 120–720 IU/day for females from 2002 to 2012 [45]. In Australia, besides food mandated for vitamin D fortification (namely edible oil spreads e.g. margarine), very few eligible products are voluntarily fortified [48]. We recently modelled vitamin D food fortification of fluid milk and alternatives using nationally-representative food consumption data and showed potential increases in vitamin D intake of about 80 IU/day among the general Australian population [48]. Our findings from this study will allow similar modelling of vitamin D fortification scenarios using commonly consumed staple foods such as bread, dairy milk, and breakfast cereals, consumed by 70%, 69%, and 34% of Aboriginal and Torres Strait Islander peoples, respectively [10]. The modelling of vitamin D fortification could support potential policy and practice decisions to promote vitamin D sufficiency across the entire Australian population.

A strength of our study was using the most comprehensive nationally representative food consumption data and comprehensive food composition data for retail food products and game products in Australia. We quantified vitamin D intake using food composition data that comprised all four D vitamers, and we used both bioactivity factors 1 and 5 for 25(OH)D to account for the higher biological activity of 25(OH)D. Our findings provide a baseline to monitor trends in vitamin D intake over time in the Aboriginal and Torres Strait Islander population.

Our study was limited by the availability of only one day of 24-h food recall for those living in remote areas [9]. As the vitamin D intake quantified from a single day is unsuitable for assessing nutrient adequacy, we could only compare usual vitamin D intakes in those living in non-remote areas to the estimated average requirement or tolerable upper intake level [49]. While the food consumption data used in this study were collected in 2012–2013, this is the first and only nationally representative nutrition-specific survey conducted among Aboriginal and Torres Strait Islander peoples. We did not include dietary supplement intake when quantifying vitamin D intake as detailed vitamin D supplement data were not collected as part of the NATSINPAS [4]. The low percentage [<1% (*n* = 38)] of supplement consumption in the 24 h prior to the study interview also suggests that supplement intake would not have a significant impact on the quantification of vitamin D intake. Lastly, despite using the Automated Multiple-Pass Method to assist respondents in providing an accurate 24-h food recall, under-reporting of dietary intake was prevalent in NATSINPAS [50], which may result in the underestimation of vitamin D intake.

We found that vitamin D intake was low among Aboriginal and Torres Strait Islander peoples in Australia. Using findings from this study, food-based public health strategies, guided by Aboriginal and Torres Strait Islander Elders and communities, could be developed to promote vitamin D sufficiency among this population group.

## Supplementary information

Supplementary file

## Data Availability

Data described in the manuscript were source from publicly available datasets found here: 2012-2013 National Aboriginal and Torres Strait Islander Nutrition and Physical Activity Survey https://www.abs.gov.au/statistics/microdata-tablebuilder/microdatadownload, AUSNUT 2011-2013 https://www.foodstandards.gov.au/science-data/monitoringnutrients/afcd/australian-food-composition-database-download-excel-files#nutrient and vitamin D food composition data https://www.foodstandards.gov.au/science-data/monitoringnutrients/afcd/Data-provided-by-food-companies-and-organisations.
