# Correction: Quantifying vitamin D intake among Aboriginal and Torres Strait Islander peoples in Australia

**DOI:** 10.1038/s41430-026-01726-1

**Published:** 2026-03-19

**Authors:** Belinda Neo, Dale Tilbrook, Noel Nannup, Alison Daly, Eleanor Dunlop, John Jacky, Carol Michie, Cindy Prior, Brad Farrant, Carrington C. J. Shepherd, Anita S. Lawrence, Edoardo Tescari, Lucinda J. Black

**Affiliations:** 1 Curtin Medical School, Curtin University, Bentley, Western Australia Australia; 2 Maalinup Aboriginal Gallery, Caversham, Western Australia Australia; 3 The Kids Institute, The University of Western Australia, Nedlands, Western Australia Australia; 4 Curtin School of Population Health, Curtin University, Bentley, Western Australia Australia; 5 Institute for Physical Activity and Nutrition (IPAN), School of Exercise and Nutrition Sciences, Deakin University, Geelong, Victoria Australia; 6 Ngangk Yira Institute, Murdoch University, Murdoch, Western Australia Australia; 7 School of Agriculture and Food, and Ecosystem Sciences, The University of Melbourne, Parkville, Victoria Australia; 8 Melbourne Data Analytics Platform, The University of Melbourne, Parkville, Victoria Australia

**Keywords:** Nutrition, Public health

Correction to: *European Journal of Clinical Nutrition* 10.1038/s41430-025-01580-7, published online 19 February 2025

The authors regret to inform the readership of a minor labelling error in the age group categories of Tables 1 and 2 and Supplementary Tables 1, 2 and 5. Specifically, age group 51–70 years should read 51–74 years, and ≥71 years should read ≥75 years. All analyses were conducted using the correct age group definitions, and this error does not affect the results or conclusions. The incorrect age group labels appear in statements in the results section, where: Older adults aged ≥ 71, should read ≥75 years had the lowest median vitamin D intake; Older female adults aged ≥ 71, should read ≥75 years had the lowest median vitamin D intake at 41 IU/day and similar to bioactivity factor 1, older adults aged ≥ 71, should read ≥ 75 years had the lowest median vitamin D intake.

