# Supplementary material for: Quantifying vitamin D intake among Aboriginal and Torres Strait Islander peoples in Australia
Source: Eur J Clin Nutr. 2025 Feb 19;79(6):529–35. doi: 10.1038/s41430-025-01580-7 (PMC12151864; doi:10.1038/s41430-025-01580-7)
Supplement: Supplementary file 1 — Supplementary file [file 41430_2025_1580_MOESM1_ESM.pdf]

Supplementary Table 1. Vitamin D intake of Aboriginal and Torres Strait Islander peoples living in non-remote areas, stratified by sex and age group.

Age group (years)	n	n[1]	Bioactivity factor 1 Percentile (IU/day)					Bioactivity factor 5[2] Percentile (IU/day)				
			5th	25th	50th	75th	95th	5th	25th	50th	75th	95th
**Male**												
All ages	797	237696	17	53	90	165	351	43	117	185	304	541
2 - 3	47	13161	16	43	52	81	222	59	88	124	171	379
4 - 8	106	33883	12	50	72	146	311	32	91	137	244	487
9 - 13	97	29465	15	62	103	169	437	43	127	184	287	472
14 - 18	83	27698	12	47	100	159	276	35	114	220	321	568
19 - 30	137	52572	25	57	97	191	372	66	136	216	385	588
31 - 50	177	52474	22	65	107	178	348	55	151	203	327	565
51 - 70	138	26721	19	46	80	140	285	38	117	188	271	517
≥ 71	12	1722	25	34	64	162	496	43	86	117	229	619
**Female**												
All ages	995	240205	13	39	74	124	275	31	86	143	240	487
2 - 3	55	11602	14	28	57	100	280	43	84	122	199	340
4 - 8	108	33175	22	45	76	114	292	44	88	145	212	491
9 - 13	79	26821	19	50	88	140	233	39	100	157	246	488
14 - 18	70	27775	8	27	52	111	356	21	66	111	256	557
19 - 30	195	50087	11	39	77	125	275	26	88	148	254	477
31 - 50	305	59881	11	40	79	126	253	28	88	152	254	453
51 - 70	162	27510	13	40	78	139	374	34	82	150	260	639
≥ 71	21	3354	22	33	41	71	125	53	89	96	129	223

[1]Weighted to the benchmark of Aboriginal and Torres Strait Islander estimated resident population living in private dwellings of Australia at 30 June 2011, based on the 2011 Census of Population and Housing, with survey weight provided by the Australian Bureau of Statistics.

[2]A bioactivity factor of 5 was used as 25-hydroxyvitamin D may be up to five times more bioactive than vitamin D.

Supplementary Table 2. Vitamin D intake of Aboriginal and Torres Strait Islander peoples living in remote areas, stratified by sex and age group.

Age group (years)	n	n[1]	Bioactivity factor 1 Percentile (IU/day)					Bioactivity factor 5[2] Percentile (IU/day)				
			5th	25th	50th	75th	95th	5th	25th	50th	75th	95th
**Male**												
All ages	1017	64296	11	48	84	145	342	34	104	174	306	613
2 - 3	78	3786	10	29	52	74	133	28	74	116	146	265
4 - 8	153	7070	15	42	68	108	246	35	91	154	196	375
9 - 13	110	8401	14	67	89	133	273	37	140	255	298	456
14 - 18	81	6395	10	51	91	173	381	26	104	160	342	636
19 - 30	162	13962	10	50	105	177	385	34	118	244	321	627
31 - 50	250	16494	15	52	85	170	389	39	120	173	331	819
51 - 70	170	7748	6	39	65	121	231	20	90	156	253	488
≥ 71	13	440	36	38	51	135	202	90	125	172	192	364
**Female**												
All ages	1300	64718	10	39	74	133	323	30	91	161	262	553
2 - 3	60	2463	7	19	33	48	198	13	62	72	126	378
4 - 8	148	8562	12	30	59	103	267	30	72	131	210	435
9 - 13	123	6243	21	45	79	125	266	61	97	138	213	404
14 - 18	98	6955	7	37	75	136	331	22	95	168	253	540
19 - 30	228	13947	15	47	95	159	386	33	112	182	339	641
31 - 50	366	17272	11	43	76	146	310	28	92	174	270	501
51 - 70	252	8679	10	46	81	121	316	35	99	173	286	478
≥ 71	25	597	10	13	25	96	239	35	37	72	180	453

[1]Weighted to the benchmark of Aboriginal and Torres Strait Islander estimated resident population living in private dwellings of Australia at 30 June 2011, based on the 2011 Census of Population and Housing, with survey weight provided by the Australian Bureau of Statistics.
[2]A bioactivity factor of 5 was used as 25-hydroxyvitamin D may be up to five times more bioactive than vitamin D.

Supplementary Table 3. Top 10 food group contributors to vitamin D intake among Aboriginal and Torres Strait Islander peoples living in non-remote areas.[1]

Bioactivity factor 1	IU	%	Bioactivity factor 5[2]	IU	%
Fats and oils[3]	39341	19.2	Meat, poultry, game products and dishes	104477	28.2
Meat, poultry, game products and dishes	36090	17.6	Egg products and dishes	58125	15.7
Biscuits, pastries, cakes, burgers, and pasta	26334	12.9	Biscuits, pastries, cakes, burgers, and pasta	48312	13.1
Egg products and dishes	24096	11.8	Milk products (e.g., milk, yoghurt, cheese, ice cream)	43284	11.7
Fish and seafood products and dishes	22720	11.1	Fats and oils[3]	40901	11.1
Non-alcoholic beverages	17187	8.4	Fish and seafood products and dishes	23932	6.5
Milk products (e.g., milk, yoghurt, cheese, ice cream)	12753	6.2	Non-alcoholic beverages	18776	5.1
Confectionery and cereal/nut/fruit/seed bars	8324	4.1	Breakfast cereals and bread	8821	2.4
Breakfast cereals and bread	7669	3.7	Confectionery and cereal/nut/fruit/seed bars	8508	2.3
Meal replacement and protein powders	3075	1.5	Vegetable products and dishes	4643	1.3

[1]Calculated as (total vitamin D intake from food group/total vitamin D intake from all foods) x 100.

[2]A bioactivity factor of 5 was used as 25-hydroxyvitamin D may be up to five times more bioactive than vitamin D.

[3]Fats and oils food group include margarine, table spreads, and butter.

Supplementary Table 4. Top 10 food group contributors to vitamin D intake among Aboriginal and Torres Strait Islander peoples living in remote areas.[1]

Bioactivity factor 1	IU	%	Bioactivity factor 5[2]	IU	%
Meat, poultry, game products and dishes	61460	23.6	Meat, poultry, game products and dishes	177427	36.1
Fats and oils[3]	50815	19.5	Egg products and dishes	101798	20.7
Fish and seafood products and dishes	42825	16.4	Fats and oils[3]	52651	10.7
Egg products and dishes	42149	16.2	Fish and seafood products and dishes	44784	9.1
Biscuits, pastries, cakes, burgers, and pasta	21240	8.1	Milk products (e.g., milk, yoghurt, cheese, ice cream)	37553	7.6
Non-alcoholic beverages	15105	5.8	Biscuits, pastries, cakes, burgers, and pasta	37034	7.5
Milk products (e.g., milk, yoghurt, cheese, ice cream)	11311	4.3	Non-alcoholic beverages	16260	3.3
Confectionery and cereal/nut/fruit/seed bars	3540	1.4	Breakfast cereals and bread	5030	1.0
Breakfast cereals and bread	2768	1.1	Reptiles, amphibia and insects	3722	0.8
Reptiles, amphibia and insects	2696	1.0	Confectionery and cereal/nut/fruit/seed bars	3638	0.7

[1]Calculated as (total vitamin D intake from food group/total vitamin D intake from all foods) x 100.

[2]A bioactivity factor of 5 was used as 25-hydroxyvitamin D may be up to five times more bioactive than vitamin D.

[3]Fats and oils food group include margarine, table spreads, and butter.

Supplementary Table 5. Usual vitamin D intake of Aboriginal and Torres Strait Islander peoples living in non-remote areas, stratified by sex and age group.

Age group (years)	n	n[1]	Bioactivity factor 1						Bioactivity factor 5[2]					
			Mean (IU/day)	Percentile (IU/day)					Mean (IU/day)	Percentile (IU/day)				
				5th	25th	50th	75th	95th		5th	25th	50th	75th	95th
**Male**														
2 - 3	47	13161	86	35	57	77	105	165	158	76	114	147	190	279
4 - 8	106	33883	110	47	74	100	134	204	187	92	136	176	225	320
9 - 13	97	29465	127	60	91	120	156	219	212	110	158	202	256	348
14 - 18	83	27698	121	56	87	114	147	211	236	122	178	225	283	387
19 - 30	137	52572	159	77	115	150	192	271	293	157	222	280	348	472
31 - 50	177	52474	140	67	100	131	170	240	253	133	190	241	302	412
51 - 70	138	26721	110	51	78	103	135	193	215	112	160	204	258	355
≥ 71	12	1722	131	59	91	122	159	230	215	106	156	203	258	360
**Female**														
2 - 3	55	11602	79	32	52	71	98	148	146	69	104	136	178	252
4 - 8	108	33175	99	42	66	90	123	184	170	83	122	160	207	290
9 - 13	79	26821	113	49	78	106	141	200	203	100	148	192	247	343
14 - 18	70	27775	87	35	59	81	109	162	169	82	121	159	206	292
19 - 30	195	50087	100	43	68	93	124	180	183	91	133	172	223	314
31 - 50	305	59881	97	41	67	91	120	175	182	90	132	171	219	309
51 - 70	162	27510	114	50	79	106	141	201	195	97	141	183	236	330
≥ 71	21	3354	70	27	46	65	87	134	133	63	95	124	159	234

[1]Weighted to the benchmark of Aboriginal and Torres Strait Islander estimated resident population living in private dwellings of Australia at 30 June 2011, based on the 2011 Census of Population and Housing, with survey weight provided by the Australian Bureau of Statistics.
[2]A bioactivity factor of 5 was used as 25-hydroxyvitamin D may be up to five times more bioactive than vitamin D.
